# Elucidating the Pivotal Immunomodulatory and Anti-Inflammatory Potentials of Chloroquine and Hydroxychloroquine

**DOI:** 10.1155/2020/4582612

**Published:** 2020-09-25

**Authors:** Seidu A. Richard, Sylvanus Kampo, Maite Esquijarosa Hechavarria, Marian Sackey, Alexis D. B. Buunaaim, Eugene Dogkotenge Kuugbee, Thomas Winsum Anabah

**Affiliations:** ^1^Department of Medicine, Princefield University, P. O. Box MA128, Ho, Ghana West Africa; ^2^Department of Anesthesia and Critical Care, School of Medicine, University of Health and Allied Sciences, Ho, Ghana; ^3^Department of Pharmacy, Ho Teaching Hospital, P.O. Box MA374, Ho, Ghana West Africa; ^4^Department of Surgery, School of Medicine and Health Science, University for Development Studies, Tamale, Ghana; ^5^Department of Clinical Microbiology, School of Medicine and Health Science, University for Development Studies, Tamale, Ghana; ^6^Department of Clinical Medicine, Habana Medical Services, Tamale, Ghana

## Abstract

Chloroquine (CQ) and hydroxychloroquine (HCQ) are derivatives of 4-aminoquinoline compounds with over 60 years of safe clinical usage. CQ and HCQ are able to inhibit the production of cytokines such as interleukin- (IL-) 1, IL-2, IL-6, IL-17, and IL-22. Also, CQ and HCQ inhibit the production of interferon- (IFN-) *α* and IFN-*γ* and/or tumor necrotizing factor- (TNF-) *α*. Furthermore, CQ blocks the production of prostaglandins (PGs) in the intact cell by inhibiting substrate accessibility of arachidonic acid necessary for the production of PGs. Moreover, CQ affects the stability between T-helper cell (Th) 1 and Th2 cytokine secretion by augmenting IL-10 production in peripheral blood mononuclear cells (PBMCs). Additionally, CQ is capable of blocking lipopolysaccharide- (LPS-) triggered stimulation of extracellular signal-modulated extracellular signal-regulated kinases 1/2 in human PBMCs. HCQ at clinical levels effectively blocks CpG-triggered class-switched memory B-cells from differentiating into plasmablasts as well as producing IgG. Also, HCQ inhibits cytokine generation from all the B-cell subsets. IgM memory B-cells exhibits the utmost cytokine production. Nevertheless, CQ triggers the production of reactive oxygen species. A rare, but serious, side effect of CQ or HCQ in nondiabetic patients is hypoglycaemia. Thus, in critically ill patients, CQ and HCQ are most likely to deplete all the energy stores of the body leaving the patient very weak and sicker. We advocate that, during clinical usage of CQ and HCQ in critically ill patients, it is very essential to strengthen the CQ or HCQ with glucose infusion. CQ and HCQ are thus potential inhibitors of the COVID-19 cytokine storm.

## 1. Introduction

Chloroquine (CQ) and hydroxychloroquine (HCQ) are derivatives of 4-aminoquinoline compounds with over 60 years of safe clinical use in the treatment of malaria and, recently, the treatment of inflammatory disorders [1, 2]. CQ and HCQ have proven to be an effective and safe treatment option for autoimmune diseases like rheumatoid arthritis (RA) as well as systemic lupus erythematosus (SLE) [1, 3]. Also, in recent years, CQ and HCQ have gained special attention because of the nonexistence of effective and efficient antiviral medications against new emerging viruses such as human immunodeficiency virus (HIV), dengue virus, chikungunya virus, and Ebola virus [4–6]. These compounds are readily available, cost effective, highly tolerated by the body, and elicit very critical immunomodulatory activities [4]. The structure and mechanism of action of CQ and HCQ are exactly the same except for an extra hydroxy moiety in one terminal in HCQ [7, 8].

After oral ingestion, CQ and HCQ are absolutely and rapidly absorbed into the blood stream [2]. The proteins in both compounds are bound in plasma and partly metabolized through the cytochrome P450 (CYP) enzymes in the liver [2]. CQ undergoes hepatic modifications via the N-dealkylation pathway into two functional metabolites such as desethyl-CQ and bisdesethyl-CQ. In human liver microsomes, CYP2C8 and CYP3A4/5 are the key enzymes accountable for the CQ N-desethylation to desethyl-CQ [2, 9]. On the other hand, HCQ is metabolized into one main metabolite, N-desethyl-HCQ, by CYP enzymes CYP2D6, CYP2C8, CYP3A4, and CYP3A5 via the N-desethylation pathway. *In vivo* studies have demonstrated a correlation between blood N-desethyl-HCQ levels and effectiveness of HCQ [2, 10].

CQ and HCQ amass in tissues with elevated levels in the liver, brain, heart, muscle, and skin than the blood after prolong usage [2, 11, 12]. Therefore, it was speculated that tissue levels may be more associated with their effectiveness than blood levels [2, 13]. Studies have demonstrated that the buildup of CQ and HCQ in lymphocytes as well as macrophages resulted in anti-inflammatory activities in diverse viral diseases depicted with the overproduction of tumor necrosis factor-*α* (TNF-*α*) by the alveolar macrophages [14, 15]. Also, CQ precisely blocked TNF, interleukin- (IL-) 6, and prostaglandin (PG) E release without modulating the expression of IL-1 by normal macrophages [16].

CQ and HCQ are capable of modulating immune players like toll-like receptors (TLRs), T-cells, B-cells, interferons (IFNs), mitogen-activated protein kinase (MAPK), chemokines, and generation of reactive oxygen species (ROS) [17–22]. This review therefore explicitly explores the key immune and inflammatory players modulated by CQ and HCQ. Most of the articles reviewed were indexed in PubMed with strict inclusion criteria being *in vitro* and *in vivo* up- or downregulation of these immune and inflammatory biomarkers in different disease conditions.

## 2. Mechanism of Action and Dosage

CQ and HCQ easily penetrate the lipid bilayer due to their small lipophilic nature after oral or intramuscular administration [23, 24]. Inside the cell, these diprotic weak bases diffuse across a pH gradient into acidic subcellular compartments like endolysosomes where they become diprotonated at lower pH [23, 25]. The diprotonated CQ and HCQ accumulates inside endolysosomes up to 10,000-fold elevations as compared to their extracellular levels leading to curatively accessible intracellular levels in the millimolar range [23, 24, 26]. These drugs inhibited protein synthesis and processing, as well as degradation via mechanisms involving alkalization of endosomes and lysosomes [23]. Nevertheless, extra actions of the drugs seem to be independent of lysosomotropism [23, 27].

Also, these drugs are capable of interrelating with DNA resulting in the modification of its super helical structure, as well as inhibition of DNA synthesis at extreme concentrations [23, 27]. Furthermore, studies have shown that they are capable of inhibiting inositol 1,4,5-triphosphate signaling as well as protein phosphorylation [23, 28]. Qu et al. demonstrated that the total ROS and mitochondrial (mt) ROS levels in QBC939 cells were obviously augmented while mitochondrial membrane potentials were obviously diminished after CQ treatment [29]. CQ and CHQ are capable of neutralizing the cellular acidic compartments containing lysosomes as well as endosomes [30–32]. They have been implicated in the modification and the intracellular trafficking of newly synthesized proteins [30–32]. HCQ can regulate human inflammatory macrophage polarization through the downregulation of M1 contrary to the upregulation of M2 macrophages [33, 34].

CQ was capable of inhibiting the function of lysosomes, leading to a widespread blockage of autophagy [29]. It was established that oxidized proteins cannot be damaged by molecular chaperone-mediated autophagy in lysosomes, which are inhibited by CQ [29]. Thus, CQ is likely to have a robust blockade effect on the antioxidant capacity and cell-death-stimulatory properties [29, 35]. Studies have demonstrated that CQ triggered a bitter taste receptor (TAS2R) leading to an upsurge in intracellular Ca^2+^ via the G*βγ*-PLC*β*-IP3-IP3R signaling pathway in the airway smooth muscle [36–38]. It was also established that the upsurge in Ca^2+^ was probably mediated in the CQ-triggered glucose transporter 4 (GLUT4) trafficking to the plasma membrane [36].

The plasma concentration of CQ peaked half an hour after administration while the plasma concentration of HCQ peaked within 3-4 hours after administration [39, 40]. A study revealed that the action of CQ and HCQ in the blockade of TNF-*α*, IL-1*β*, and IL-6 synthesis operated via diverse approaches and their therapeutic doses were capable of suppressing the production of TNF-*α*, IL-1*β*, and IL-6 in patients [30]. The intracellular concentrations of HCQ in mononuclear cells from patients on a 3-month course of standard dose of 400 mg daily was similar to that in mononuclear cells incubated *in vitro* with 100 *μ*M CQ or HCQ for 1 hour [26, 30]. CQ and HCQ have long terminal as well as elimination half-lives of 22 and 20-60 days, respectively [39, 40]. Nevertheless, the excretion HCQ in the urine persists up to 3 months from the time of the last dose [39].

CQ and HCQ are mostly in tablet formula for oral usage as CQ phosphate 500 mg which is equivalent to 300 mg CQ base and HCQ sulfate 200 mg which is equivalent to 155 mg HCQ base active drug per tablet, respectively [39]. It is advocated that, in autoimmune diseases like rheumatoid arthritis and systemic lupus erythematosus, the doses of CQ and HCQ should not exceed 500 mg/day and 400 mg/day, respectively [39]. Nevertheless, in acute malaria, doses as high as 2000 mg CQ and HCQ have been used [39, 40]. We are of the view that these drugs may target the protozoan cells with less affinity for normal body cells in malaria, thus reducing the adverse effects of the drugs due to high concentrations in the protozoan cells and lesser concentration in the normal body cells.

In viral as well as autoimmune diseases, the drugs may target only the normal body cells, thereby increasing adverse effects with doses exceeding 500 mg/day for CQ and 400 mg/day for HCQ. Complications like retinopathy and QTc prolongation with consequential possibility of ventricular arrhythmias have been associated with CQ and HCQ [39]. CQ has higher possibilities of retinopathy than HCQ though short-term dosing of both medicines has no such complication [39, 41].

## 3. Toll-Like Receptor (TLR)

The key mechanism of action of HCQ is the blockade of nucleic acid-sensing toll-like receptors (TLRs) [17]. The inhibitory effect of nucleic acid-sensing TLRs occurs as a result of reduction in endosomal pH or direct binding of nucleic acid machineries to the TLRs [17–19]. In both *in vitro* and *in vivo* experimental prototypes, CQ inhibited proinflammatory cytokine secretion triggered by microbial TLR ligands via downregulating TLR-9 and TLR-4 mRNA secretion, inhibiting NF-*κ*B as well as activated protein-1 (AP-1) stimulation, interfering with endosome maturation, and blockade of nucleic acid binding to TLR-7, TLR-8, and TLR-9 (Table 1) [42, 43]. Studies have shown that CQ was capable of triggering endosomal acidification and fusion, thus blocking the stimulation and facilitation of the virus via endocytic TLR-3, TLR-7, TLR-8, and TLR-9 (Table 1) in HIV patients [20, 21, 44]. Also, CQ was capable of inhibiting TLR-7 downregulatory signaling pathways resulting in the blockade of transcription factors like interferon regulatory factor- (IRF-) 7, which modulates the production of IFN-*α*, an effective CD8 T-cell immune stimulator [20–22].

Plasmacytoid dendritic cells (pDC), which identify pathogens via TLR-7 and TLR-9, are an essential component of the innate and adaptive immune systems [22]. TLRs are intracellular, and thus, their ligands involve cellular uptake as well as endosomal maturation to trigger NF-*κ*B and MAPK-mediated signals via the MyD88-dependent pathway. These TLR signals result in pDC stimulation or maturation and in the generation of proinflammatory cytokines as well as huge quantities of IFNs-*α*/*β* [22]. Martinson et al. demonstrated that CQ blocked pDC stimulation or maturation, upregulation of the MyD88 pathway signaling molecules like IRF-7 and IL-1 receptor-associated kinase 4 (IRAK-4), IFN-*α* generation, indoleamine 2,3-dioxygenase (IDO) synthesis, and programmed death-ligand 1(PDL-1) secretion. The precise association between these markers and CQ or HCQ in viral disease still needs further studies [22].

## 4. Interferons

IFNs have been implicated in several immune responses as triggers and modulators as well as effectors of both innate and adaptive immune systems during viral infections [45, 46]. IFNs have the capability of inhibiting viral replication and are often the most conspicuous cytokines produced during viral infections [45, 46]. Studies have shown that the concentrations of IFN and IFN-inducible chemokines/cytokines like macrophage inflammatory protein-1 (MIP-1) and monocyte chemotactic protein-1 (MCP-1) as well as interferon-inducible protein-10 (IP-10) are associated with disease burden [47–49]. These chemokines or cytokines are measured by the different disease activity indices, the erythrocyte sedimentation rate, and anti-dsDNA antibody titers [47–49].

Cytokine and TCR-triggered IFN-*γ* secretion was via separate signal transduction pathways comprising of transcription factors such as nuclear factor of activated T-cells (NFATs), signal transducer and activator of transcriptions (STATs), and nuclear factor kappa-light-chain-enhancer of activated B-cells (NF-*κ*B) [45]. This resulted in the stimulation as well as the triggering of several intrinsic antiviral factors like RNA-activated protein kinase (PKR), the 2-5A system, Mx proteins, and many apoptotic pathways [45]. Studies have shown that human IFN-*α* and IFN-*γ* bound to receptors and entered cells via receptor-mediated endocytosis through coated pits as well as endosomes [50, 51]. Studies have indicated that IFN acted from outside the cell membrane to attain antiviral state [50, 52, 53]. Studies have further proven that the antiviral activity was triggered when IFNs bind to an insoluble matrix and the nonactivity of IFN microinjected directly into cells [50, 54, 55].

Type I IFN offers effective innate immune machinery against a verity of viruses, but it may also stimulate pathogenic immune response, thus leading to huge loss of stimulated CD4^+^ T-cells. Branca et al. reported that CQ induced the production of the 2′,5′-A synthetase [56]. Nevertheless, Chelbi-Alix and Thang found out that the presence of CQ during IFN treatment does not affect the triggering of the 2′,5′-A synthetase but impairs the IFN-dependent inhibition of virus growth [50]. Studies have demonstrated that CQ and HCQ inhibited the production of IFN-*α* and IFN-*γ* and/or TNF-*α* (Table 1) [50]. Studies using different cell populations have demonstrated that HCQ inhibited proinflammatory cytokines, like TNF-*α*, IFN-*γ*, IL-1*α*, and IL-6 (Table 1) [50, 57–59]. Also, HCQ inhibited the production of IFN-*α* (Table 1) in pDCs *in vitro*, either after stimulation by DNA-containing immune complexes or upon activation with TLR-9 agonists [60]. The explicit pathways via which CQ and HCQ trigger the release or inhibit the release of IFNs still need further studies since current evidence is paradoxical.

## 5. Interleukins

Several studies have persistently shown an inhibition of the production of cytokines such as IL-1, IL-2, IL-6, IL-17, and IL-22 (Table 1) by CQ and HCQ [30, 61]. In RA patients, IL-1 was primarily produced by monocytes and macrophages in the synovial tissue and was strongly implicated in joint destruction [30, 62]. Studies have demonstrated that a huge quantity of IL-1*β* perhaps in the form of pro-IL-1*β* was retained in cells and the concentration of cell-associated IL-1*β* was reduced by CQ (Table 1) [30, 63]. This study further revealed that the blockade of IL-1*β* production stimulated by weak-base amines occurred via the inhibition of pro-IL-1*β* rather than via reduced IL-1*β* mRNA [30]. Studies have demonstrated that CQ was capable of inhibiting the release of IL-1*β* via a pathway involving endolysosome-associated vesicles in lipopolysaccharide- (LPS-) stimulated monocytes [30, 64].

CQ was capable of inhibiting IL-2 generation and IL-2 mRNA stimulation as well as the alteration of IL-2 receptiveness of T-cell clones [65]. Studies have shown that IL-2 generation by *α*CD3 MoAb-triggered T-cells was possibly modulated in an autocrine fashion [65–67]. It was affirmed that *α*CD3 MoAb triggers the generation of IL-2, and IL-2 stimulated T-cell proliferation via the interrelation of IL-2 with high affinity IL-2R [65]. Nevertheless, CQ did not affect secretory levels of the IL-2R p55 chain, but it is possible that it might influence some other component of the IL-2 receptor (IL-2R) complex [65, 68]. It was established that the interrelation between IL-2 and its receptor resulted in augmentation of IL-2 generation during CQ administration [65, 67]. Also, blockade of lL-2 generation as well as blockade of T-cell proliferation led to either rescindment of the triggering signal or blockade of IL-2 receptiveness [65]. Landewe et al. demonstrated that CQ partly blocks the internalization and completely inhibited the intracellular degradation of IL-2 (Table 1) [65].

IL-6 is a pleiotropic cytokine produced by macrophages and T-cells as well as synovial fibroblasts in inflammatory joint tissues [30, 69]. IL-6 facilities synovitis by triggering antibody production due to its influence on B-cell maturation. It also triggered T-cells as well as stimulated the proliferation of synovial fibroblasts [30, 70]. CQ was capable of inhibiting IL-6 synthesis (Table 1) in LPS-stimulated mouse macrophages as well as human monocytes, though the mode of blockage was dissimilar in mouse and human cells [16, 30, 58, 71]. Studies with human peripheral blood mononuclear cells (PBMCs) demonstrated that CQ decreased LPS-induced secretion of IL-1*β* as well as IL-6 mRNA [23, 30, 72]. Yu et al. found that the role of HCQ in decreasing plasma IL-6 (Table 1) concentrations was highly coherent with the length of its administration, and once the medicine was halted, plasma IL-6 concentrations reverted to the control concentrations [33]. They demonstrated that HCQ was capable of mimicking the influential properties of anti-IL-6 antibody by significantly decreasing the concentrations of IL-6 in the critically ill COVID-19 patients [33].

Jang et al. observed a decreased in IL-1*β* and IL-6 (Table 1) release after treatment of PBMCs and monocytes or macrophages with CQ [30]. CQ blocked IL-1*β* and IL-6 generation via reduction of their mRNA levels, resulting in reduction in mRNA stability rather than alteration of transcriptional activity [30]. They indicated further that CQ regulated some steps involved in the synthesis as well as metabolism of IL-1*β* and IL-6 mRNA [30]. These steps included transcription of IL-1*β* and IL-6 genes, the processing of prime transcripts in the nucleus, the transport of processed mRNA to the cytosol, and the degradation of mRNA [30]. In their nuclear run-on analysis, transcriptional behaviors of the IL-1*β* and IL-6 genes in LPS-stimulated monocytes or macrophages were not considerably transformed by CQ, signifying that CQ did not influence the synthesis of key transcripts of these cytokines [30].

Cruz da Silva et al. demonstrated a blockade of IL-17 and IL-22 (Table 1) supernatant levels by HCQ. They indicated that HCQ reduced helper T-cell (Th) 17 cytokine levels in the PBMCs from healthy individuals and SLE or RA patients [57]. It was established that IL-17 augmented the immune reaction by augmenting target organ inflammation as well as damage. Also, IL-17 enhances antibody production by B-cells, a significant immune player in SLE [73]. The exact mechanism via which HCQ decreased IL-6 and IL-17 as well as IL-22 concentrations is still a matter of debate. Nevertheless, one potential explanation is that the reduction occurred via decreasing Th17 cells through a reduction in antigen presentation [57].

## 6. T-Cells

Helper T-cells (Th) are phenotypically heterogeneous in nature [57, 74]. They are categorized based on the cytokines they generated via the innate immune system during the process of Th-cell differentiation. Th1, Th2, Th17, and regulatory T-cells (Treg) are 4 principal lineages described [57, 74]. In periphery, Treg cells are capable of triggering self-reactive lymphocytes via cell contact and expression of anti-inflammatory cytokines as well as alteration of proficient antigen presenting cells, such as DCs [75–78]. Studies have shown that adoptive transfer of Treg cells decreased inflammatory diseases, like human graft versus host disease, experimental arthritis, experimental autoimmune hepatitis, experimental diabetes, and experimental autoimmune encephalomyelitis [75]. Therefore, Treg cells are suitable for the decrease of chronic inflammation perceived in most autoimmune diseases [75].

The stimulation of T-cells occurred via the triggering of T-cell receptors [65]. It was well established that stimulated T-cells triggered IL-2 mRNA resulting in the production of IL-2 protein [65]. The expressed IL-2 in turn triggered T-cell proliferation via binding to IL-2R present on activated T-cells [65]. Landewe et al. indicated that the inhibition of T-cell proliferation by CQ means that CQ stimulated the alteration of receptor-mediated endocytosis [65]. Studies have shown that HCQ is capable of inhibiting Treg cell-induced upregulation of CD69 [79, 80]. Nevertheless, HCQ failed to elicit inhibitory effect during evaluation of multiple proximal Treg cell-mediated signaling events such as Treg cell-induced protein tyrosine-kinase stimulation, inositol phosphate generation, and MAPK stimulation [79]. CQ affected the stability between Th1 and Th2 cytokine secretion by augmenting IL-10 production in PBMC [1, 81].

Several studies have demonstrated that HCQ in human immunodeficiency virus/acquired immunodeficiency syndrome (HIV/AIDS) patients stabilized CD4 T-cell counts or elevation (Table 1) when used in combination with hydroxyurea as well as didanosine [20, 82–85]. Piconi et al. demonstrated that 6-month HCQ therapy in combination with antiretroviral therapy (ART) was associated with reduced immune stimulation as well as augmented CD4^+^ T-cell frequency [86]. Routy et al. demonstrated contrary findings compared to the Piconi et al. findings (Table 1) [20]. Routy et al. detected reduced secretion of the maturation marker CD83 on pDCs after CQ therapy, which they assumed possibly contributed to a reduction in DC-mediated inflammation [20].

A study revealed that both untreated and CQ-treated animals suffered a deep loss of CD4^+^ T-cells during the acute phase of infection [87]. Nevertheless, the ability to regenerate peripheral CD4^+^ T-cells was obviously enhanced initially and subsequently hindered by CQ therapy in the long term [87]. CQ therapy during chronic simian immune deficiency virus infection exhibited a decrease in immune stimulation as well as an enhanced recovery of CD4^+^ T-cells, but this did not influence virus levels [87, 88]. The precise pathways via which CQ and HCQ influence CD4^+^ T-cells still need further studies since current evidence is inconsistent.

## 7. B-Cells

Clusters of differentiation (CD) 19^+^ B-cells are categorized into 3 functionally definite subsets: immunoglobulin (Ig) D^+^CD27^−^ naïve B-cells, IgD^+^CD27^+^ IgM memory B-cells, and IgD^−^CD27^+^ class-switched memory B-cells [3, 17]. Class-switched memory B-cells proliferate in the peripheral blood as well as inflammatory tissues of patients with extremely vigorous RA or SLE and are linked to the worsening of these autoimmune diseases [17, 89–91]. There are 5 classes of immunoglobulins generated by antibody-producing cells during disease process. IgG is the most effective inflammation facilitator, due to its robust antigen-binding affinity and complement-activation as well as opsonic capability [17, 92]. Thus, IgGs produced by self-reactive B-cells are presumed to perform pathogenic autoantibody functions [17].

Studies have shown that HCQ inhibited extreme autoimmune responses as well as exerts therapeutic effects by blocking the ligation of TLRs with nucleic acids [17, 93–95]. It was established that nucleic acid-sensing TLRs are secreted by human B-cells [17, 93, 94]. These TLR ligations activated B-cells to regulate inflammatory responses via antibody and cytokine production, as well as antigen presentation [17, 93–95]. Torigoe et al. showed that HCQ at clinical levels effectively blocked CpG-triggered class-switched memory B-cells from differentiating into plasmablasts as well as producing IgG [17]. Also, HCQ also inhibited cytokine generation from all the B-cell subsets (Table 1) [17]. IgM memory B-cells exhibited the utmost cytokine production [17].

Torigoe et al. found that TLR-9 secretion was predominantly elevated in resting B-cells and CpG activation more effectively triggered B-cells to proliferate as well as differentiate into plasmablasts compared to loxoribine activation [17]. They indicated that the extremely secreted TLR-9 could sensitively identify dsDNA-containing antigens and were extremely capable of facilitating the inflammatory responses of B-cells in infection prevention as well as autoimmune diseases [17]. Studies have demonstrated that CQ decreased the survival of CpG-activated B-cells and suppressed the secretion of coactivators as well as blocked the facilitatory effect of IL-10 production (Table 1) [17, 96, 97]. Cepika et al. also affirmed that IgM memory B-cells exhibited the utmost effective cytokine-generating capability as compared to class-switched memory B-cells and naïve B-cells and HCQ competently blocked all the three B-cell subsets from producing inflammatory cytokines (Table 1) [96].

## 8. Prostaglandins

Prostaglandins (PGs) are produced in numerous types of tissue injury as well as acute and chronic inflammation [98, 99]. The levels of exogenous PGs produced during inflammatory response often reproduced as well as augmented the cardinal signs of inflammation such as edema, erythema, and hyperalgesia [98, 99]. Floman demonstrated that CQ blocked the production of PGs (Table 1) in the intact cell by inhibiting substrate accessibility of arachidonic acid necessary for the production of PGs [98]. Floman further indicated that CQ may decrease arachidonic acid accessibility via the blockade of phospholipase A2 activity [98]. *In vitro* studies using a murine hemorrhagic shock model were inconclusive on the inhibitory effect of CQ on cytokines as well as PG synthesis and the lowering of Kupffer cell function like antigen presentation and I*α* secretion [16, 100]. Contrarily, CQ and HCQ inhibited IL-1, IL-2, IL-6, IL-17, and IL-22 as well as PGs (Table 1) [61, 98, 99].

## 9. Tumor Necrotizing Factor

Tumor necrosis factor (TNF) is a pleiotropic cytokine that partakes in crucial regulatory roles in immune and inflammatory responses via cell surface receptors [59, 101]. Studies have identified p55 and p75 as the 2 distinctive categories of TNF receptors (TNF-R) amongst members of the TNF-R superfamily [59, 102]. It was affirmed that the p55 TNF-R was secreted universally on the surface of almost all cell types, whereas the p75 TNF-R was secreted predominantly in hematopoietic cells as well as endothelial cells [59]. It was established that both TNF-R possess four common cysteine-rich extracellular domains via which they bind TNF with high affinity [59]. Also, the cytoplasmic regions on both receptors are different and transmit distinctive but interrelating signals. These receptors have been implicated in the stimulation of nuclear factor beta (NF-*κ*B) as well as TNF-mediated apoptosis [59, 101, 103].

Studies revealed that p55 TNF-R intermediated in TNF signals in lethal endotoxaemia as well as nonspecific immunity to infection, whereas p75 TNF-R inhibits TNF­mediated inflammatory responses during gene knockout [59, 104, 105]. It was well established that CQ triggered downregulation of cell surface p75 TNF-R in human peripheral blood monocytes incubated with phorbol 12-myristate 13-acetate and/or BB-3103 [59, 105]. Nevertheless, in resting monocytes, the blockade effect of CQ was not observed, probably because resting monocytes exhibited low levels of TNF-R secretion [59]. It was further affirmed that cell surface p75 TNF-R considerably increased when receptor shedding was inhibited by BB-3103 but was partially blocked by CQ [59]. CQ also reduced the surface secretion of TNF-R in inactivated cells in a similar degree as was seen in the protein expression blocker monensin and brefeldin A [59]. Thus, CQ inhibited soluble TNF-R generation by blocking the intracellular trafficking of these molecules to the cell surface, instead of inhibiting cleavage of TNF-R on the cell surface [59].

Studies have demonstrated that TNF-*α* was crucial for the development of both the innate as well as the adaptive immune response [23, 106]. It was affirmed that neutralization of TNF-*α* with mAbs or soluble TNF-*α* receptors resulted in enhanced clinical outcomes in certain infectious and autoimmune diseases [23, 107]. TNF-*α* was capable of modulating posttranslations at the transcriptional level. It was established that, after translation, the 26 kDa membrane-bound pro-TNF-*α* was cleaved at the cell surface by a matrix metalloproteinase, TNF-*α* converting enzyme (ADAM-17), freeing a soluble 17 kDa form of the cytokine [23, 108]. It was proven that secretion of the antigen-presenting process occurred in parallel with an augmented TNF secretion by Kupffer cells as well as obvious augmentation of circulating TNF levels 2 hours after hemorrhage [16].

Monocytes and macrophages are the main source of TNF-*α* during RA pathogenesis [23, 30, 107, 109]. CQ was capable of inhibiting TNF-*α* synthesis (Table 1) in LPS-activated mouse macrophages as well as human monocytes, though the blockade route was dissimilar in the mouse and human cells [16, 30, 58, 71]. CQ blocked TNF-*α* synthesis via inhibiting the conversion of cell-mediated TNF-*α* precursor to the soluble mature form, rather than blocking the stimulation of TNF-*α* mRNA or synthesis of TNF-*α* precursor (Table 1) [30, 109]. Studies with human PBMCs revealed that CQ decreased LPS-triggered secretion of TNF-*α*, as well as cell-related TNF-*α* [23, 30, 72]. Jang et al. demonstrated that CQ blocked TNF-*α* secretion (Table 1) but did not alter the level of TNF-*α* mRNA or the synthesis of TNF-*α* precursor [30]. They indicated that blockade of TNF-*α* synthesis by CQ occurred at a posttranslational step rather than a transcriptional step [30]. Also, the blockade effect of CQ on TNF-*α* synthesis occurred at a step in the processing of pro-TNF-*α* as well as the release of mature proteins [30].

## 10. Mitogen-Activated Protein Kinase

The extracellular signal-regulated kinases (ERK) 1/2 are meticulously necessitated for TNF transcription in some human and murine macrophage populations, whereas p38 and the c-Jun N-terminal kinase (JNK) are meticulously necessitated for posttranscriptional modulation of TNF synthesis [110–113]. It was well affirmed that ERK was stimulated via a serine-threonine kinase cascade activated by Raf phosphorylation of the ERK activating kinases like MAP/ERK kinases (MEK) 1/2 [110]. Furthermore, Raf stimulation was activated via recruitment of this protein to the membrane by the protooncogene Ras resulting in Raf phosphorylation [110, 114]. Nevertheless, the phosphorylation of Raf at Ser259 led to inactivation of this enzyme [110, 115]. Therefore, phosphorylation at different domains led to an up- or downregulation of this signaling pathway [110]. The Raf-MEK-ERK signaling was very essential in a wide range of macrophage inflammatory activities [110].

Weber et al. demonstrated that CQ precisely inhibited the stimulation of ERK-MAP kinase proteins (Table 1) which are obligatory for prime LPS-triggered TNF secretion in human mononuclear phagocytes and murine macrophage cell line AMJ2C-8 [23, 110, 116]. CQ was also capable of blocking LPS-triggered stimulation of extracellular signal-modulated ERK1/2 in human PBMCs (Table 1) [30]. Moreover, the secretion of the TNF-*α* promoter-driven reporter gene in human monocytic THP-1 cells revealed that CQ inhibited the transcription of the TNF-*α* gene via blockade of LPS-triggered stimulation of the ERK1/2 signaling pathway [30, 110]. Further *in vitro* and *in vivo* studies on this pathway and CQ are still warranted.

## 11. Chemokines

Chemokines are a group of molecules implicated in the trafficking of leukocytes in normal immune surveillance as well as recruitment of inflammatory cells in host defense [1, 117, 118]. They comprise over 40 members, which are categorized into four classes based on the locations of fundamental cysteine residues such as C, CC, CXC, and CX3C [1]. CQ was capable of stimulating the mRNA and protein levels of chemokines like CCL2 and CXCL8 in human astroglial cells [1]. The stimulation of these chemokine mRNAs was detected at 3 hours, optimum at 16 hours, and persisted up to 24 hours after CQ therapy [1]. It was speculated that the upsurge in mRNA secretory levels of these proinflammatory chemokines was as a result of either transcriptional stimulation or stabilization of mRNA by CQ [1].

It was affirmed that CQ therapy resulted in stimulation of CXCL8 promoter activities (Table 1), which means that transcriptional stimulation was partially accountable for mRNA secretion of chemokines [1]. It was established that CQ triggered stimulation of the NF-*κ*B transcription factor, and blockade of NF-*κ*B stimulation inhibited CQ-triggered chemokine secretion in astroglial cells [1]. This strongly indicates that stimulation of chemokines was mediated at the transcriptional level [1].

Studies have demonstrated that leukocytes and neutrophils as well as eosinophils secreted CXCL10 during inflammation [119, 120]. Also, monocytes, epithelia, endothelial, and stromal cells as well as keratinocytes are expressed in response to IFN-*γ* during inflammation [119, 121, 122]. Th1 cells generated IFN-*γ*, which stimulates the CXCL10 production by diverse cell types [119]. CXCL10 in turn attracted and recruited Th1 cells, signifying the occurrence of a positive feedback loop between IFN-*γ*-producing Th1 cells and resident cells producing CXCL10 [119, 123]. Further studies on the effects of CQ or HCQ on the positive feedback loop between IFN-*γ* producing Th1 cells and resident cells producing CXCL10 in viral diseases are warranted.

## 12. Reactive Oxygen Species

Cells produce reactive oxygen species (ROS) via metabolism and respiratory burst, as well as the respiratory chain [29]. Cells clear ROS through peroxisomes, superoxide dismutase, and the nicotinamide adenine dinucleotide phosphate- (NADPH-) dependent reduction system, as well as the autophagy-lysosome pathway resulting in the regulation of reduction-oxidation (REDOX) balance in cells [29, 124]. Several extracellular stimuli have been implicated in the stimulation of the transient upsurge in intracellular ROS levels [1, 125]. Also, inhibition of intracellular ROS led to a substantial blockade of stimulant-dependent signaling in mammalian cells [1, 125]. In the REDOX balance adjustment process, the principal source of mitochondrial (mt) ROS was oxidative respiration. Studies have demonstrated that disruption of mitochondrial functions was capable of augmenting mtROS generation as well as triggering cell death [29, 124, 126].

Park et al. demonstrated that ROS generated by CQ facilitated the stimulation of NF-*κ*B following the secretion of chemokines in human astroglial cells (Table 1) [1]. Nevertheless, they observed that CQ did not trigger an upsurge of intracellular ROS in human monocytic U937 cells and murine microglial BV-2 as well as macrophage RAW 264.7 cells. They indicated that disparities in the immunomodulatory effect of CQ between monocytes, microglia, and astroglial cells seem to be determined at the level of ROS production following the stimulation of NF-*κ*B [1]. It was affirmed that CQ-triggered production of ROS was annulled by diphenyl iodonium, signifying the probability that nonphagocytic NADPH oxidase partook in the production of ROS during CQ therapy [1]. Qu et al. demonstrated that the overall ROS and mtROS levels in QBC939 cells were augmented severely after CQ administration (Table 1) while mitochondrial membrane potentials were severely reduced [29]. They concluded that CQ was capable of triggering an upsurge in ROS level (Table 1), specifically mtROS, in QBC939 cells which resulted in the loss of mitochondrial membrane potentials [29].

## 13. Glucose Metabolism

Glucose uptake is primarily reliant on GLUT4 which translocates extracellular glucose via the cell membrane into the cell [36, 127]. Therefore, GLUT4 is very critical for sustenance whole-body glucose homeostasis [36, 127]. It was well established that GLUT4 was predominantly located in intracellular GLUT4-storage vesicles (GLUT4-SVs) [127]. Studies have shown that insulin triggered fast translocation of GLUT4SVs from the trans-Golgi network and/or endosomes to the plasma membrane [36, 128]. The fusion of GLUT4-SVs with the plasma membrane led to augmented glucose uptake [128]. Further studies have demonstrated that this step was upregulated via insulin receptor or insulin receptor substrate-1(IRS-1), protein kinase B (PKB/Akt), phosphatidylinositol 3 kinase (PI3-K), and atypical protein kinase C (aPKC) as well as cytosolic Ca^2+^ [129, 130].

Studies revealed that CQ facilitated cellular glucose uptake via the stimulation of GLUT4 trafficking to, and fusion with (Table 1), the cellular plasma membrane via augmentation of cellular Ca^2+^ uptake [36, 131, 132]. Another study demonstrated that CQ was an effective stimulator of the insulin-responsive protein like PKB/Akt and considerably augmented glycogen synthesis via the phosphorylation of glycogen synthase kinase 3*β* (GSK-3*β*), which made it an attractive potential antidiabetic drug [36, 133]. It was further established that the antidiabetic mechanism of CQ analogues involved reductions in insulin clearance as well as degradation rates and an upsurge in the expression of C-peptide [134, 135].

CQ and HCQ are well-tolerated therapeutic options for type II diabetic mellitus [134]. Glycated hemoglobin reduced considerably when HCQ was combined with insulin for the treatment of diabetes mellitus, compared with patients receiving placebo, and the insulin dose had to be lowered by 30% in the HCQ group [134, 136]. We anticipate that, in critically ill patients, CQ and HCQ are likely to deplete all the energy stores of the body leaving the patient very weak and sicker. A study revealed that a rare, but serious, side effect of CQ or HCQ in nondiabetic patients is hypoglycaemia (Table 1) [132, 137]. Thus, during clinical usage of CQ or HCQ in critically ill patients, it is very essential to strengthen the CQ or HCQ with glucose infusion.

## 14. Conclusion

CQ and HCQ are able to inhibit the production of cytokines such as IL-1, IL-2, IL-6, IL-17, and IL-22. Also, CQ blocked TNF-*α* synthesis via inhibiting the conversion of cell-mediated TNF-*α* precursor to the soluble mature form, rather than blocking the stimulation of TNF-*α* mRNA or synthesis of TNF-*α* precursor. Furthermore, CQ was also capable of inhibiting IL-2 generation and IL-2 mRNA stimulation as well as the alteration of IL-2 receptiveness of T-cell clones. Similarly, HCQ also inhibits cytokine generation from all the B-cell subsets. IgM memory B-cells exhibit the utmost cytokine production. Nevertheless, CQ is capable of producing ROS via a facilitated stimulation of NF-*κ*B and following secretion of chemokines in human astroglial cells. A rare, but serious, side effect of CQ or HCQ in nondiabetic patients is hypoglycaemia. We advocate that, in critically ill patients, CQ and HCQ are more likely to deplete all the energy stores of body leaving the patient very weak and sicker. Thus, during clinical usage of CQ or HCQ in critically ill patients, it is very essential to strengthen the CQ or HCQ with glucose infusion. CQ and HCQ are thus potential inhibitors of the COVID-19 cytokine storm.

## Figures and Tables

**Table 1:** Shows the explicit effect of CQ or HCQ on various immune/inflammatory factors.

Immune/inflammatory factors	Type	Effect of CQ/HCQ	Citation
Toll-like receptors (TLRs)	TLR-3	Inhibition	[20, 21, 44]
	TLR-7	Inhibition	[20, 21, 42–44]
	TLR-8	Inhibition	[20, 21, 42–44]
	TLR-9	Inhibition	[20, 21, 44]

Interferons (IFNs)	IFN-*α*	Inhibition	[50, 57–59]
	IFN-*β*	Inconclusive	No data
	IFN-*γ*	Inhibition	[50, 57–59]

Interleukins	IL-1	Inhibition	[30, 61–63]
	IL-2	Inhibition	[30, 61, 65, 67, 68]
	IL-6	Inhibition	[16, 23, 30, 33, 58, 61, 70–72]
	IL-10	Facilitator	[1, 81]
	IL-17	Inhibition	[30, 57, 61]
	IL-22	Inhibition	[30, 57, 61]

T-cells	Th1	Stability	[1, 81]
	Th2	Stability	[1, 81]
	Th17	Inconclusive	No data

CD4^+^	Inconclusive	[20, 82–85]
B-cells	All B-cell subsets	Inhibition	[17, 93–95]

Prostaglandins (PGs)	PGs	Inhibition	[98, 99]

Tumor necrosis factor (TNF)	TNF-*α*	Inhibition	[16, 23, 30, 58, 71, 72, 109]

Extracellular signal-regulated kinases (ERK)	1/2	Inhibition	[23, 30, 110, 116]

Chemokines	CXCL8	Facilitator	[1]

Reactive oxygen species (ROS)	—	Facilitator	[1, 29]

Glucose	—	Facilitator	[36, 131, 132, 134, 136, 137]

## Abbreviations

AP-1: Activated protein-1
aPKC: Atypical protein kinase C
CD: Cluster of differentiation
CQ: Chloroquine
CYP: Cytochrome P450
ERK1/2: Extracellular signal-regulated kinases
GLUT4: Glucose transporter 4
GLUT4SVs: GLUT4 storage vesicles
HIV: Human immunodeficiency virus
HCQ: Hydroxychloroquine
IL: Interleukin
IL-2R: IL-2 receptor
IRF: Interferon regulatory factor
IFN: Interferon
IRS-1: Insulin receptor substrate-1
JNK: c-Jun N-terminal kinase
MAPK: Mitogen-activated protein kinase
mt: Mitochondrial
NFATs: Nuclear factor of activated T-cells
NF-*κ*B: Nuclear factor kappa-light-chain-enhancer of activated B-cells
NADPH: Nicotinamide adenine dinucleotide phosphate
PI3-K: Phosphatidylinositol 3 kinase
pDC: Plasmacytoid dendritic cells
PGs: Prostaglandins
PKR: Protein kinase
PBMCs: Peripheral blood mononuclear cells
ROS: Reactive oxygen species
RA: Rheumatoid arthritis
REDOX: Reduction-oxidation
Treg: Regulatory T-cells
SLE: Systemic lupus erythematosus
STATs: Signal transducer and activator of transcriptions
Th: T-helper cell
TNF: Tumor necrotizing factor
TLRs: Toll-like receptors.
